# Anisotropic magnetic coupling with a two-dimensional characteristic in noncentrosymmetric Cr_11_Ge_19_

**DOI:** 10.1038/srep39338

**Published:** 2016-12-20

**Authors:** Hui Han, Lei Zhang, Xiangde Zhu, Haifeng Du, Min Ge, Langsheng Ling, Li Pi, Changjin Zhang, Yuheng Zhang

**Affiliations:** 1 High Magnetic Field Laboratory, Chinese Academy of Sciences, Hefei 230031, China; 2 University of Science and Technology of China, Hefei 230026, China; 3 Hefei National Laboratory for Physical Sciences at the Microscale, University of Science and Technology of China, Hefei 230026, China

## Abstract

In this work, we successfully synthesize the single crystal Cr_11_Ge_19_. The magnetism of the noncentrosymmetric Cr_11_Ge_19_ with itinerant ferromagnetic ground state is thoroughly investigated on the single crystal. Based on the variation measurements including the angular rotation, temperature, and magnetic field dependence of magnetization, we find that this material exhibits strong magnetic anisotropy along the *c*-axis. To clearly reveal the magnetic interactions, the critical behavior is studied using the modified Arrott plot, the Kouvel-Fisher method, and the critical isotherm technique. Combining these different methods, three main critical exponents (*β, γ*, and *δ*) are obtained. The critical exponent *β* is close to the theoretical prediction of a three-dimensional XY model with spin-dimensionality *n* = 2, indicating two-dimensional magnetic coupling. Meanwhile, the critical exponent *γ* suggests that the magnetic interaction is of long-range type with magnetic exchange distance decaying as J(*r*) ≈ *r*^−4.61^. We propose that the ferromagnetic ground state of Cr_11_Ge_19_ is formed by the polarized magnetic moments along the *c*-axis, while the long-range magnetic coupling is established within the *ab* plane.

Itinerant ferromagnets, which are characterized by low saturation moment and Curie temperature, have been extensively studied due to their exotic physical phenomena such as superconductivity, quantum critical behavior, non-Fermi-liquid behavior, and unusual magnetic excitation^1^,^2^,^3^,^4^,^5^,^6^,^7^. According to the band theory of electrons, magnetic moments in these systems stem from the exchange splitting of bands^8^. Because of the extremely small band splitting, the saturation moment is only a fraction of a Bohr magneton, which is very close to the nonmagnetic phase boundary. Therefore, a small perturbation can arouse a large change to the electronic and magnetic properties^9^,^10^. The thermodynamical properties of itinerant ferromagnets have usually been explained by conventional Stoner-Wohlfarth theory of band ferromagnetism based on Hartree-Fock mean-field theory^11^. On the other hand, the theoretical model based on the self-consistent renormalization considering spin fluctuations is found to be more satisfactory for describing the electronic and magnetic behaviors in these systems^12^,^13^. Recently, noncentrosymmetric itinerant ferromagnets exhibiting chiral magnetic ordering have attracted considerable attention because of the discovery of magnetic particle-like configurations, such as skyrmion, magnetic soliton, and chiral bobber^14^,^15^,^16^,^17^. The lack of an inversion center in noncentrosymmetry usually results in Dzyaloshinsky-Moriya (DM) interaction, which is 1~2 orders of magnitude smaller than that of the ferromagnetic coupling. The competition between the DM interaction and ferromagnetic coupling often results in non-collinear magnetic ordering states such as helimagnetism, conical ordering state, and magnetic particle-like configurations. Due to the extra perturbation brought by the noncentrosymmetry, the investigation of noncentrosymmetric itinerant ferromagnet is of great importance in understanding the exotic phenomena in these systems.

The noncentrosymmetric Cr_11_Ge_19_, which exhibits tetragonal structure with the space group 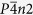, is crystallized belonging to a family of compounds known as Nowotny chimney ladders^18^. A chiral structure of Cr-Cr bonds arranges along the *c*-axis. Due to the noncentrosymmetric characteristic, the DM interaction may exist in Cr_11_Ge_19_, which makes it a candidate possessing a magnetic particle-like configuration^19^. The Cr_11_Ge_19_ exhibits complex ferromagnetic ground state^20^,^21^,^22^. Early work has demonstrated that the ground state of Cr_11_Ge_19_ is a *p*-type semi-metallic ferromagnetism^22^. However, a linear muffin tin orbital (LMTO) calculation of electronic density of states has suggested it to be an itinerant ferromagnet with low magnetic moment^23^. Recent studies have manifested that Cr_11_Ge_19_ displays complex itinerant ferromagnetism. However, the magnetic behavior cannot be explained by the Stoner model which is a conventional theory describing the itinerant ferromagnet^24^. Both the experimental results and calculations have indicated that Cr_11_Ge_19_ is a good example of an itinerant electron ferromagnet, with signatures of spin wave excitation and magnetic fluctuation^24^.

In this work, we successfully synthesize the single crystal Cr_11_Ge_19_. The magnetism of Cr_11_Ge_19_ on the single crystal is investigated. We find that the magnetization of Cr_11_Ge_19_ exhibits strong magnetic anisotropy along the *c*-axis in the ferromagnetic phase. Moreover, the study of critical behavior suggests that the magnetic interaction is of long-range type with two-dimensional magnetic coupling.

## Results and Discussion

**Figure 1:**
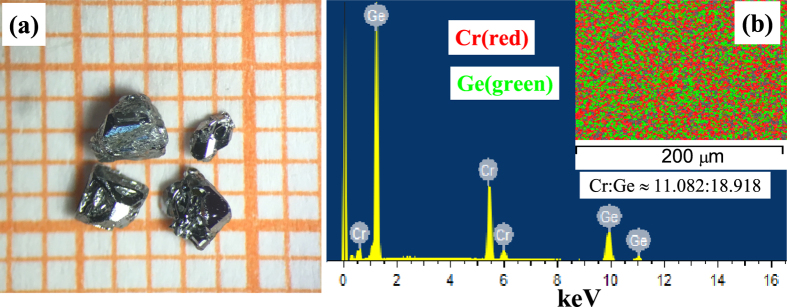
(**a**) The photograph of Cr_11_Ge_19_ single crystals; (**b**) a typical EDX spectrum for single crystal Cr_11_Ge_19_ (the inset shows the distribution of elements).

**Figure 2:**
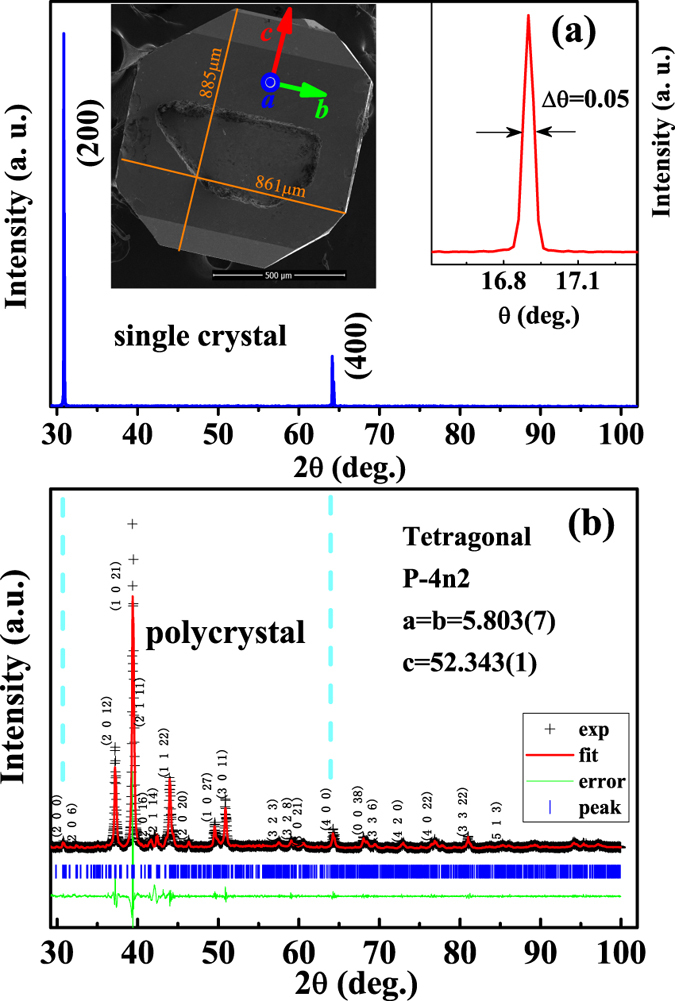
(**a**) The XRD pattern of the surface at room temperature for the single crystal (the left inset shows the morphology of the single crystal; the right inset gives the rock curve of (200) plane) (**b**) the XRD pattern at room temperature for powder Cr_11_Ge_19_.

Figure 1(a) presents the photograph of Cr_11_Ge_19_ single crystals, which shows that the typical sizes of these single crystals are in millimeter scale. The shapes of the single crystals are three-dimensional graininess with small bright surfaces. Figure 1(b) gives a typical EDX spectrum of the Cr_11_Ge_19_ single crystal. The EDX spectra measured at different points indicate that the proportion of Cr:Ge is close to 11:19 (Please see the Supplementary Information). The inset of Fig. 1 gives the distribution of the elements, which indicates that Cr and Ge are distributed homogeneously. The left inset of Fig. 2(a) depicts the morphology of a typical single crystal with size of 885 × 861 *μ*m. The bright surface was checked by XRD as shown in Fig. 2(a), which indicates that the surface is (200) plane. The right inset of Fig. 2(a) presents the rock curve of the (200) diffraction peak. The full-width-at-half-maximum (FWHM) of the rock curve is Δ*θ* = 0.005°. The single peak and narrow FWHM of the rock curve indicate high quality of the single crystal sample without twin crystal. The other crystal orientations in the *bc* plane are determined by Laue photograph. The crystal orientations are marked on the single crystal in the left inset of Fig. 2(a). Figure 2(b) shows the XRD pattern for powder Cr_11_Ge_19_ ground from several pieces of single crystals. The powder XRD pattern also indicates a single phase of Cr_11_Ge_19_ without impurities. The fitting results give the lattice constants *a* = *b* = 5.803(7) *Å* and *c* = 52.343(1) *Å*, which is in agreement with previous reports^20^,^22^,^24^.

**Figure 3:**
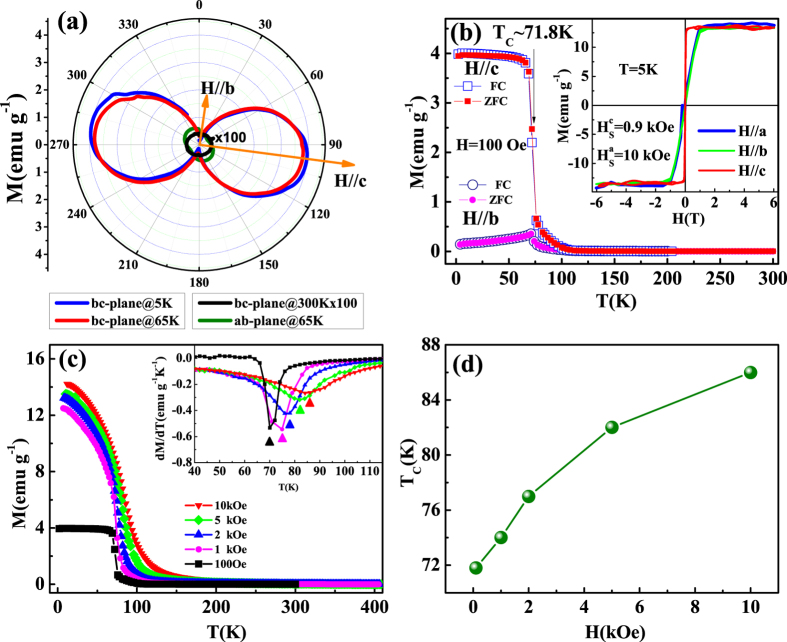
(**a**) The magnetization as a function of rotation angel [*M*(*φ*)]; (**b**) the temperature dependence of magnetization [*M*(*T*)] with the isothermal magnetization [*M*(*H*)] at 5 K in the inset; (**c**) *M*(*T*) curves along the *c*-axis under different *H* (the inset gives the *dM*/*dT* vs. *T*); (**d**) the field dependence of *T*_*C*_ determined from the minimum from *dM*/*dT* curves.

Figure 3(a) gives the magnetization as a function of rotation angle [*M*(*φ*)] under *H* = 100 Oe. The *M*(*φ*) curves in both *ab* and *bc* planes are measured. In the *bc* plane, it can be seen that the value *M* along the *c*-axis is much larger than that along the *b*-axis at temperatures of 65 K and 5 K, revealing strong magnetic anisotropy along the *c*-axis. However, there is no magnetic anisotropy at 300 K. In the *ab* plane, much weaker magnetic anisotropy is observed at 65 K. The variation of *M*(*φ*) curves indicates that Cr_11_Ge_19_ displays strong magnetic anisotropy, and the easy magnetization orientation is along the *c*-axis. It is noted that the cell of Cr_11_Ge_19_ is tetragonal with equal *a* and *b* axes, while the Nowotny chimney ladders are found along the *c*-axis^24^. Therefore, the exhibition of strong magnetic anisotropy along the *c*-axis corresponds closely to the tetragonal crystal structure. Figure 3(b) displays the temperature dependence of magnetization [*M*(*T*)] under zero-field-cooling (ZFC) and field-cooling (FC) with *H* = 100 Oe along *c*- and *b*-axis. The *M*(*T*) curves with *H*//*c* exhibit a typical paramagnetic-ferromagnetic transition. The *M*(*T*) curves with *H*//*b* are different from those with *H*//*c*. The values of *M* are much smaller and the transition becomes un-obvious when *H*//*b*. This result implies that the paramagnetic-ferromagnetic transition is mainly determined by the magnetization along the *c*-axis. The inset of Fig. 3(b) displays the isothermal magnetization [*M*(*H*)] at 5 K with *H*//*a, H*//*b*, and *H*//*c*. The *M*(*H*) curves with *H*//*a* and *H*//*b* are well overlapped with each other, which become saturated when *H* exceeds ~10 kOe. However, for *M*(*H*) curve with *H*//*c*, the situation magnetic field *H*_*S*_ (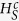 ~ 0.9 kOe) is much smaller than that with *H*//*a* or *H*//*b* (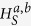 ~ 10 kOe). When *H* exceeds 10 kOe, the three *M*(*H*) curves overlap with each other reaching the same saturated moment *M*_*S*_. The obtained *M*_*S*_ is 0.43 *μ*_*B*_/Cr, which is in agreement with the previous report in a polycrystalline sample^24^. The strong magnetic anisotropy only appears when *H* is weaker than *H*_*S*_. When *H* exceeds *H*_*S*_, spins of all directions are polarized along the orientation of applied field *H*, regardless of *H*//*a, H*//*b*, or *H*//*c*. This is a process of spin rotation from unsaturated state to saturated one. The transition temperature *T*_*C*_ ~ 71.8 K is determined from the sharpest point of the phase transition, as shown in Fig. 3(b). Generally, *T*_*C*_ is actually difficult to be determined from magnetism because it is usually dependent on the external field. Figure 3(c) shows the *M*(*T*) curves under different fields with *H*//*c*, and the inset gives the *dM*/*dT* vs. *T*. The field dependence of *T*_*C*_ determined from the minimum from *dM*/*dT*(*T*) curves is depicted in Fig. 3(d), which shows that *T*_*C*_ increases monotonously with the increase of *H*. The *T*_*C*_ is ~86 K when *H* = 10 kOe, which is close to that of 86 K reported by V. L. Zagryazhskii, *et al*.^22^ and 88 K by N. J. Ghimire, *et al*.^24^ from the polycrystalline sample.

**Figure 4:**
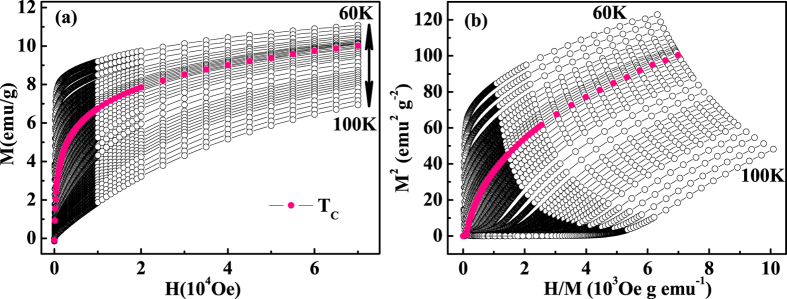
(**a**) The isothermal initial magnetization measured along the *c*-axis around *T*_*C*_ for Cr_11_Ge_19_; (**b**) the Arrott plot of *M*^2^ vs. *H*/*M*.

According to the Stoner model, the itinerant ferromagnet should follow^25^,^26^,^27^:


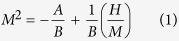


where *A* and *B* are parameters independent of *H*. Thus, the Arrott plot of *M*^2^ vs. *H*/*M* can be constructed, where the *M*^2^ vs. *H*/*M* relations should present a series of straight lines around *T*_*C*_^28^. Meanwhile, *M*^2^ vs. *H*/*M* at *T*_*C*_ should just pass through the origin^28^. Figure 4(a) shows the initial isothermal *M*(*H*) curves around *T*_*C*_ with *H*//*c (i. e*. the easy magnetization axis), and Fig. 4(b) gives the Arrott plot of *M*^2^ vs. *H*/*M* for Cr_11_Ge_19_. All curves of the Arrott plot show nonlinear behaviors even in the high field region, which suggests that the critical behavior of Cr_11_Ge_19_ cannot be described by the conventional Stoner model. The order of the phase transition can be determined by the slope from the Arrott plot according to the Banerjee’s criterion, where a negative slope corresponds to a first-order transition and a positive slope suggests a second-order one^29^. The positive slopes of *M*^2^ vs. *H*/*M* curves reveal that the phase transition in Cr_11_Ge_19_ is of a second order. However, the nonlinear of the *M*^2^ vs. *H*/*M* curves indicates that the conventional Arrott plot is invalid for Cr_11_Ge_19_.

For a second order magnetic phase transition, the magnetic interaction can be identified by the investigation of critical behavior. In the vicinity of the ferromagnetic transition, the spontaneous magnetization *M*_*S*_ and initial susceptibility *χ*_0_ can be described by a series of functions^30^,^31^:


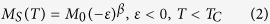



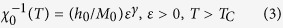



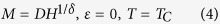


where *ε* = (*T* − *T*_*C*_)/*T*_*C*_ is the reduced temperature; *M*_0_/*h*_0_ and *D* are critical amplitudes. The parameters *β* (associated with *M*_*S*_), *γ* (associated with *χ*_0_), and *δ* (associated with *T*_*C*_) are the critical exponents. The critical behavior around the critical temperature can be described by a series of critical exponents, which follow the Arrott-Noakes equation of state in asymptotic critical region^32^:


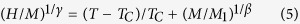


The critical exponents give significant clues about the magnetic interactions, such as the correlating length, spin-dimensionality, and decaying distance of magnetic coupling.

**Figure 5:**
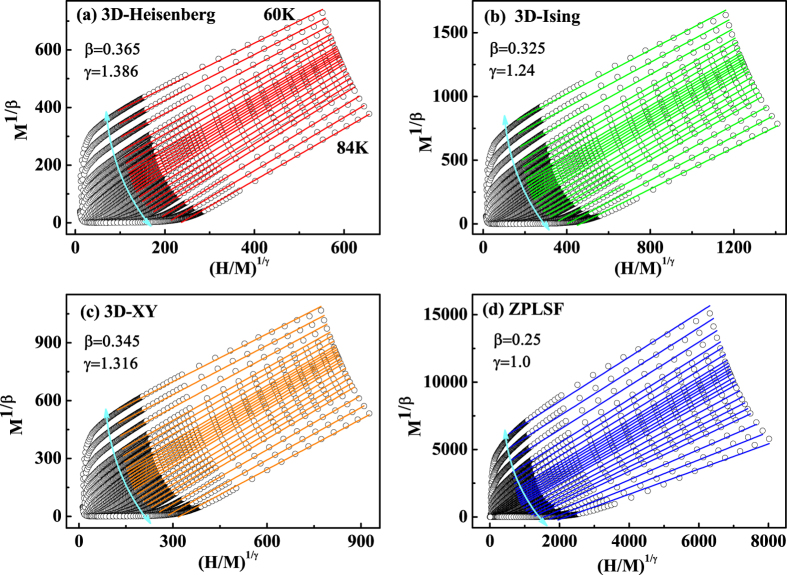
The isotherms of *M*^1/*β*^ vs. (*H*/*M*)^1/*γ*^ with parameters of (**a**) 3D-Heisenberg model, (**b**) 3D-Ising model, (**c**) 3D-XY model, and (**d**) the theoretical model considering zero point local spin fluctuation (ZPLSF).

**Figure 6:**
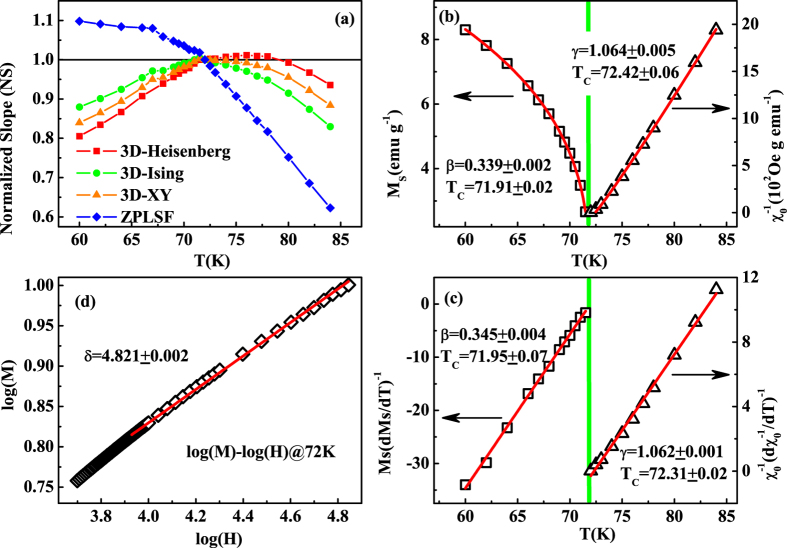
(**a**) Temperature dependence of normalized slope (*NS*); (**b**) the *M*_*S*_ (left) and *χ*_0_^−1^ (right) as a function of temperature for Cr_11_Ge_19_; (**c**) the Kouvel-Fisher plot for *M*_*S*_(*T*) (left) and *χ*_0_^−1^(*T*) (right); (**d**) isothermal *M*(*H*) at *T*_*C*_ with log-log scale (all red solid curves are fitted).

In view of the dissatisfaction of conventional Arrott plot for Cr_11_Ge_19_, a modified Arrott plot (MAP) of *M*^1/*β*^ vs. *H*/*M*^1/*γ*^ could be employed. Three kinds of modes belonging to the 3D-Heisenberg model (*β* = 0.365, *γ* = 1.336), 3D-Ising model (*β* = 0.325, *γ* = 1.24), and 3D-XY model (*β* = 0.345, *γ* = 1.316) are tried to construct the modified Arrott plots^33^,^34^, as shown in Fig. 5(a–c) respectively. According to the theoretical model suggested by Takahashi considering the zero point local spin fluctuation (ZPLSF), *M*^4^ vs. *H*/*M* should exhibit straight lines in high field region (*i*.*e. M*^1/*β*^ vs. *H*/*M*^1/*γ*^ with *β* = 0.25, *γ* = 1.0)^13^,^24^. Therefore, *M*^4^ vs. *H*/*M* curves are plotted in Fig. 5(d). All the curves in these four constructions exhibit quasi-straight lines in high field region. However, the lines in Fig. 5(d) are not parallel to each other, indicating that the theoretical model suggested by Takahashi is not satisfied for Cr_11_Ge_19_. For Fig. 5(a–c), it is difficult to distinguish which model is the best. For an ideal model, the modified Arrott plot should display a series of parallel lines in high field region with the same slope, where the slope is defined as *S*(*T*) = *dM*^1/*β*^/*d*(*H*/*M*)^1/*γ*^. The normalized slope (*NS*) is defined as *NS* = *S*(*T*)/*S*(*T*_*C*_), which enables us to distinguish the most suitable model by comparing the *NS* with the ideal value of ‘1’^35^,^36^,^37^. Plots of *NS* vs. *T* for the four different models are shown in Fig. 6(a). It can be seen that the *NS* of 3D-Heisenberg model is close to ‘1’ mostly above *T*_*C*_, while that of 3D-Ising model is the best below *T*_*C*_. This result indicates that the critical behavior of Cr_11_Ge_19_ does not belong to a single universality class.

The precise values of the critical exponents *β* and *γ* can be obtained by an iterative method^38^. The linear extrapolation from the high field region to the intercepts with the axes *M*^1/*β*^ and (*H*/*M*)^1/*γ*^ yields reliable values of *M*_*S*_(*T*, 0) and 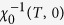. A set of *β* and *γ* values can be obtained by fitting data to Eqs (2) and (3). These obtained *β* and *γ* values are used to reconstruct a new modified Arrott plot. Subsequently, new *M*_*S*_(*T*, 0) and 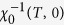 are generated from the linear extrapolation from the high field region. Therefore, another set of *β* and *γ* can be yielded. This procedure is repeated until *β* and *γ* do not change. By this method, the obtained critical exponents are hardly dependent on the initial parameters. The final *M*_*S*_(*T*, 0) and 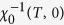 are plotted as a function of temperature in Fig. 6(b). Consequently, exponents *β* = 0.339 ± 0.002 with *T*_*C*_ = 71.92 ± 0.02 K and *γ* = 1.064 ± 0.006 with *T*_*C*_ = 72.42 ± 0.06 K are obtained.

Alternatively, critical exponents can be determined by the Kouvel-Fisher (KF) method^39^:


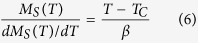



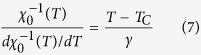


It can be seen that the *M*_*S*_(*T*)/*dM*_*S*_(*T*)/*dT* and 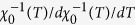 are as linear functions of temperature with slopes of 1/*β* and 1/*γ* respectively. Figure 6(c) plots the *M*_*S*_(*T*)/[*dM*_*S*_(*T*)/*dT*] and 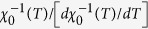 vs. *T* relations, which give more actual exponents *β* = 0.345 ± 0.004 with *T*_*C*_ = 71.95 ± 0.07 K and *γ* = 1.062 ± 0.001 with *T*_*C*_ = 72.31 ± 0.02 K. The critical exponent *δ* can be calculated by fitting to critical isothermal (CI) magnetization *M*(*H*) at *T*_*C*_ following Eq. (4). Figure 6(d) shows the *M*(*H*) at *T*_*C*_ = 72 K on log-log scale, which gives that *δ* = 4.821 ± 0.002.

**Figure 7:**
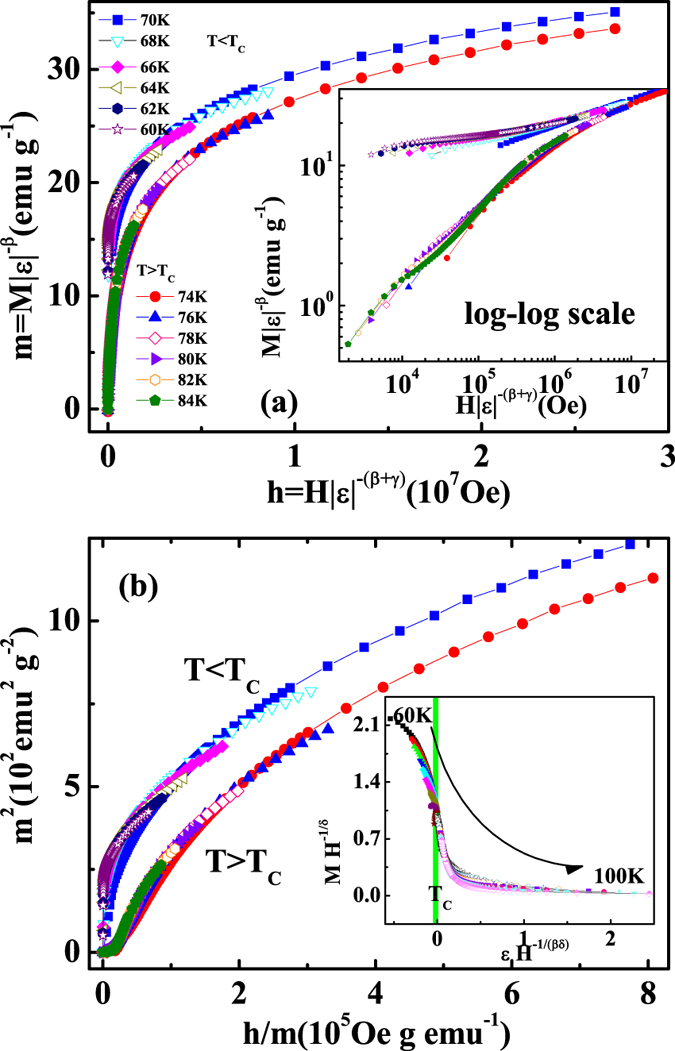
Scaling plots of *m* vs. *h* around *T*_*C*_; (**b**) *m*^2^ vs. *h*/*m* (the inset shows the re-scaling of the *M*(*H*) curves by *MH*^−1/*δ*^ vs. *εH*^−1/*βδ*^).

These critical exponents should follow the scaling equations. In the asymptotic critical region, the scaling equations can be written as^31^:


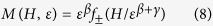


where *f*_±_ are regular functions denoted as *f*_+_ for *T* > *T*_*C*_ and *f*_−_ for *T* < *T*_*C*_. Defining the renormalized magnetization *m* ≡ *ε*^−*β*^*M*(*H, ε*), and the renormalized field *h* ≡ *Hε*^−(*β*+*γ*)^, the scaling equations indicate that *m* vs. *h* forms two universal branches for *T* > *T*_*C*_ and *T* < *T*_*C*_, respectively^40^,^41^. Based on the scaling equation [*m* = *f*_±_(*h*)], the isothermal magnetization around the critical temperatures for Cr_11_Ge_19_ are replotted in Fig. 7(a), with log-log scale in the inset. It can be seen that all experimental data, including those in low field region, collapse into two universal curves. Meanwhile, the *m*^2^ vs. *h*/*m* curves also collapse into two independent branches as shown in Fig. 7(b). Furthermore, the scaling equation of state takes another form^31^,^41^:


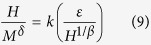


where *k*(*x*) is the scaling function. Equation (9) suggests that all experimental curves plotted on *MH*^−1/*δ*^ vs. *εH*^−1/(*βδ*)^ will collapse into a single one^41^. The inset of Fig. 7(b) shows the *MH*^−1/*δ*^ vs. *εH*^−1/(*βδ*)^ for Cr_11_Ge_19_, where the experimental data collapse into a single curve, and *T*_*C*_ locates at the zero point of the horizontal axis. The well scaling and collapse of the curves demonstrate the reliability of the experimentally obtained exponents. Generally, for a single theoretical model, the critical exponents should fulfill the Widom scaling law *δ* = 1 + *γ*/*β* according to statistical theory^42^. For Cr_11_Ge_19_, it is calculated that *δ* = 4.078 ± 0.004 according to the Widom scaling law, which slightly deviates from that obtained from the experiment. The slightly deviation from the Widom scaling law indicates that the critical behavior of Cr_11_Ge_19_ does not belong to a single universality class, which may be caused by the complex competition of several magnetic interactions. In this case of an itinerant ferromagnet, this deviation may be attributed to the discrepancy of *γ* caused by the length scale of the interaction^43^. Actually, these critical exponents are experimentally convergent, which can be confirmed by the effective exponents *β*_*eff*_ and *γ*_*eff*_ obtained as ref. 44:


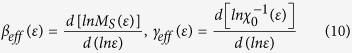


**Figure 8:**
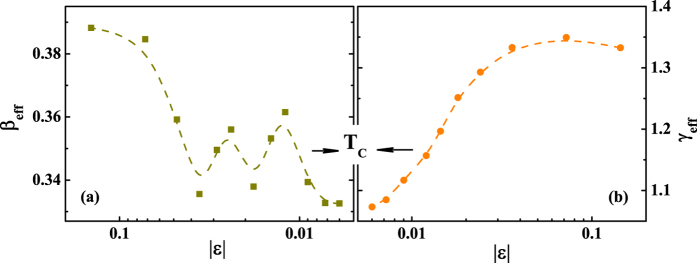
Effective exponents (**a**) *β*_*eff*_ and (**b**) *γ*_*eff*_ as a function of the reduced temperature *ε* for Cr_11_Ge_19_ (dashed curves are guided on eye).

The *β*_*eff*_ and *γ*_*eff*_ vs. *ε* for Cr_11_Ge_19_ are plotted in Fig. 8. It can be seen that *β*_*eff*_ and *γ*_*eff*_ are convergent when temperature approaching *T*_*C*_.

The obtained critical exponents of Cr_11_Ge_19_, as well as those of different theoretical models and related itinerant ferromagnetic materials, are listed in Table 1 for comparison. For a magnetic material, in addition to the spatial-dimensionality (*d*) of the crystal symmetry, the spin-dimensionality (*n*) also plays an important role in determination of the magnetic behavior^45^,^46^. For Cr_11_Ge_19_, due to the three-dimensional characteristic of its crystal structure, it gives that the spatial-dimensionality *d* = 3. However, for the spin-dimensionality, it is determined by the magnetic interaction. The critical exponent *β* of Cr_11_Ge_19_ approaches exactly that of the 3D-XY model, which means that *d* = 3 and *n* = 2^47^. It can be seen that *d* = 3 is in agreement with the structural characteristic of Cr_11_Ge_19_. Meanwhile, that *n* = 2 suggests a two-dimensional magnetic coupling. The value *γ* is close to the mean-field model, meaning a long-range magnetic interaction. The long-range magnetic interaction deduced from *γ* is in agreement with the characteristic of an itinerant ferromagnet, such as MnSi^48^, AlCMn_3_^35^, and Y_2_Ni_7_^43^. In fact, *γ* in Y_2_Ni_7_ approaches the theoretical prediction of 2D-Ising model with long-range coupling with *d* = 2 and *n* = 1 (*γ* = 1.392)^43^,^46^.

As we know, for a homogeneous magnet, the universality class of the magnetic phase transition depends on the exchange distance J(*r*). M. E. Fisher *et al*. have theoretically treated this kind of magnetic ordering as an attractive interaction of spins. Subsequently, according to the renormalization group theory, the long-range interaction decays with distance *r* as refs 49,50:


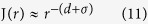


where *d* is the spatial-dimensionality and *σ* is a positive constant. Moreover, there is refs 46,50:


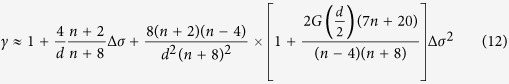


where 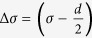 and 
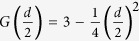
, *n* is the spin-dimensionality. For a three-dimensional material (*d* = 3), we have J(*r*) ≈ *r*^−(3+*σ*)^. When *σ* ≥ 2, the Heisenberg model is valid for the three-dimensional isotropic magnet, where J(*r*) decreases faster than *r*^−5^. When *σ* ≤ 3/2, conditions for the mean-field model are satisfied, expecting that J(*r*) decreases slower than *r*^−4.5^. From Eq. (12), it is calculated that *σ* = 1.6054 ± 0.004 when *n* = 2. Finally, we yield *J*(*r*) ≈ *r*^−4.61^, indicating a long-range magnetic coupling. Moreover, the correlation length critical exponent is obtained as *ν* = 0.662 ± 0.004 (where *ν* = *γ*/*σ, ξ* = *ξ*_0_|(*T* − *T*_*C*_)/*T*_*C*_|^−*ν*^).

**Figure 9:**
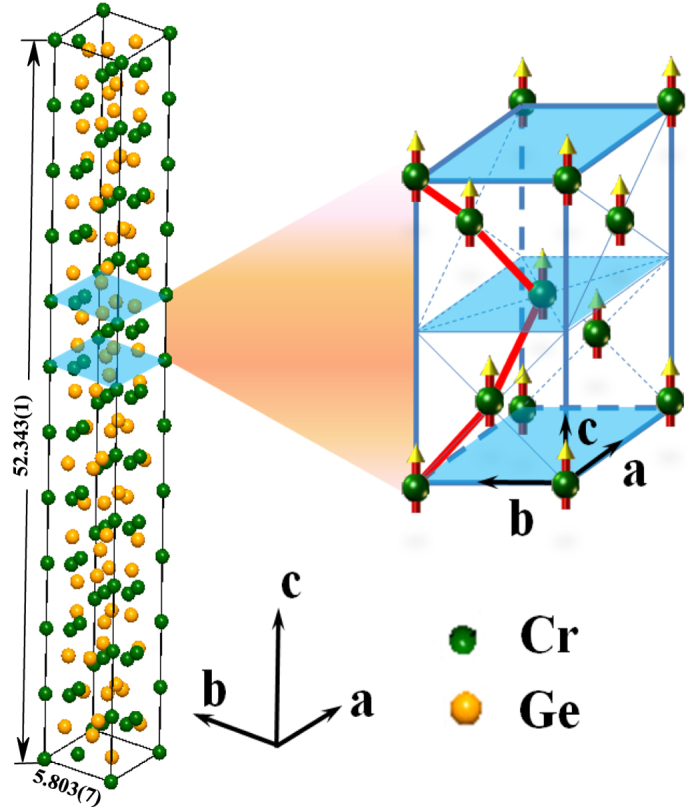
The chiral structure of Cr-Cr bonds and the magnetic structure for Cr_11_Ge_19_.

Figure 9 shows the crystal and magnetic structure for Cr_11_Ge_19_. Due to the tetragonal crystal cell of Cr_11_Ge_19_, *a* and *b* directions are equivalent in structure. Moreover, as mentioned above, *a*- and *b*-axis are also equivalent in magnetism, as shown in Fig. 3. Due to the 3D-XY characteristic, it can be concluded that the two-dimensional magnetic interaction should be within the *ab* plane, which means that the magnetic coupling strength exhibits relation *J*_*a*_ = *J*_*b*_ < *J*_*c*_. This is analogous to a two-dimensional material, where the in-plane coupling is much stronger than that of the inter-layers. However, for Cr_11_Ge_19_, it is not a two-dimensional material but a three-dimensional network. Due to the anisotropy of the Nowotny chimney ladders structure, Cr_11_Ge_19_ exhibits very strong magnetic anisotropy along the *c*-axis [see Fig. 9]. The present experimental results indicate the magnetic coupling in *ab* plane is much stronger than that along the *c*-axis. The investigation of magnetism suggests that the complex magnetic structure of Cr_11_Ge_19_ correlates closely with the crystal structure. The strong correlation between the magnetic and crystal structure should response the complex magnetic behavior in Cr_11_Ge_19_, indicating strong magneto-elastic coupling in this system. Moreover, the magneto-elastic coupling has also been confirmed by the larger abnormal change of lattice parameters in the ferromagnetic state^24^.

It is meaningful to compare the magnetic behavior of Cr_11_Ge_19_ with itinerant ferromagnets with noncentrosymmetry. Recently, a chiral magnetic soliton state has been revealed in layered Cr_1/3_NbS_2_ with noncentrosymmetry^51^. The magnetic coupling in layered Cr_1/3_NbS_2_ is demonstrated within the *ab* plane^52^, which is analogous to that in Cr_11_Ge_19_. However, the magnetic moments in Cr_1/3_NbS_2_ are arranged helically within the *ab* plane^53^, which is different with that in Cr_11_Ge_19_. On the other hand, the itinerant ferromagnetic characteristic of Cr_11_Ge_19_ is similar to that of noncentrosymmetric MnSi which exhibits a skyrmion state^48^. However, the crystal structure of MnSi is cubic, which does not exhibit so strong crystalline anisotropy as that in Cr_11_Ge_19_. The investigation of magnetism in noncentrosymmetric Cr_11_Ge_19_ indicates that it is special among the itinerant ferromagnets.

## Conclusion

In summary, the magnetism of Cr_11_Ge_19_ has been thoroughly investigated on single crystal. The angular rotation, temperature, and magnetic field dependence of magnetization [*M*(*φ*), *M*(*T*), and *M*(*H*)] display that the Cr_11_Ge_19_ exhibits strong magnetic anisotropy and the easy magnetization orientation is along the *c*-axis. Based on the study of the critical behavior, reliable critical exponents (*β, γ*, and *δ*) are obtained. The critical exponent *β* is close to the theoretical prediction of the 3D-XY model, which indicates two-dimensional magnetic coupling. The critical exponent *γ* suggests that the magnetic coupling is of long-range type, and that the magnetic exchange distance decays as J(*r*) ≈ *r*^−4.61^. We suggest that the ferromagnetic ground state of Cr_11_Ge_19_ is formed by the polarized magnetic moments along the *c*-axis, while the long-range magnetic coupling is established within the *ab* plane.

## Methods

A single crystal Cr_11_Ge_19_ was prepared by the chemical vapor transport (CVT) method. The elementary substance pieces of chromium and germanium in mol ratio of 45:55 and about 50 mg iodine were put in an evacuated quartz tube with inner diameter of 15 mm and length of about 200 mm. The tube was then placed in a two zone furnace. After heating at 1053 K for 4 days, the source end was kept at 1053 K and growth zone was raised to 1153 K. After 10 days growth, crystals in millimeter size can be obtained.

The chemical compositions were carefully checked by Energy Dispersive X-ray (EDX) spectrometry. The crystal structure was confirmed by the Rigaku-TTR3 X-ray diffractometer using high-intensity graphite monochromatized Cu K*α* radiation. The crystal orientations were determined by the Laue photography and X-ray diffraction (XRD). The magnetization was measured using a Quantum Design Vibrating Sample Magnetometer (SQUID-VSM). The no-overshoot mode was applied to ensure a precise magnetic field. The magnetic field was relaxed for two minutes before data collection. For the measurement of initial isothermal magnetization, the sample was firstly heated adequately above *T*_*C*_ for ten minutes, then cooled to the target temperature under zero magnetic field. Then the initial isothermal magnetization was performed with the magnetic field parallel to the *c*-axis (Please see the Supplementary Information).

## Additional Information

**How to cite this article:** Han, H. *et al*. Anisotropic magnetic coupling with a two-dimensional characteristic in noncentrosymmetric Cr_11_Ge_19_. *Sci. Rep.* **6**, 39338; doi: 10.1038/srep39338 (2016).

**Publisher's note:** Springer Nature remains neutral with regard to jurisdictional claims in published maps and institutional affiliations.

## Supplementary Material

Supplementary Material

## Figures and Tables

**Table 1:** Comparison of critical exponents of Cr_11_Ge_19_ with different theoretical models and other itinerant ferromagnets (MAP = modified Arrott plot; KF = Kouvel-Fisher method; CI = critical isothermal fitting).

Composition	Technique	Ref.	*TC*(K)	*β*	*γ*	*δ*
Cr_11_Ge_19_	MAP	This work	71.91 ± 0.02	0.339 ± 0.002	1.064 ± 0.005	—
Cr_11_Ge_19_	KF	This work	71.95 ± 0.07	0.345 ± 0.004	1.062 ± 0.001	—
Cr_11_Ge_19_	CI	This work	72	—	—	4.821 ± 0.002
3D-Heisenberg	theory	33	—	0.365	1.386	4.80
3D-XY	theory	33	—	0.346	1.316	4.81
3D-Ising	theory	33	—	0.325	1.241	4.82
Mean-field	theory	33	—	0.5	1.0	3.0
ZPLSF	theory	13	—	0.25	1.0	5.0
MnSi	MAP	48	30.5	0.242 ± 0.006	0.915 ± 0.003	4.734 ± 0.006
AlCMn_3_	KF	35	288.5	0.606 ± 0.009	1.177 ± 0.008	2.971 ± 0.002
YNi_7_^2*D*−*Ising*^	MAP	43	53	0.306 ± 0.002	1.401 ± 0.002	5.35

## References

[b1] Shimizu M. Itinerant electron magnetism. Rep. Prog. Phys. 44, 329 (1981).

[b2] Uhlarz M., Pfleiderer C. & Hayden S. M. Quantum Phase Transitions in the Itinerant Ferromagnet ZrZn_2_. Phys. Rev. Lett. 93, 256404 (2004). 15697921 10.1103/PhysRevLett.93.256404

[b3] Manyala N. . Agnetoresistance from quantum interference effects in ferromagnets. Nature (London) 404, 581 (2000). 10766236 10.1038/35007030

[b4] Pfleiderer C., Julian S. R. & Lonzarich G. G. Non-Fermi-liquid nature of the normal state of itinerant-electron ferromagnets. Nature (London) 414, 427 (2001). 11719799 10.1038/35106527

[b5] Watanabe H., Parameswaran S. A., Raghu S. & Vishwanath A. Anomalous Fermi-liquid phase in metallic skyrmion crystals. Phys. Rev. B 90, 045145 (2014).

[b6] Pfleiderer C., McMullan G. J., Julian S. R. & Lonzarich G. G. Magnetic quantum phase transition in MnSi under hydrostatic pressure. Phys. Rev. B 55, 8330 (1997).

[b7] Saxena S. S. . Superconductivity on the border of itinerant-electron ferromagnetism in UGe_2_. Nature (London) 406, 587 (2000). 10949292 10.1038/35020500

[b8] Blanco J. A. & Pisonero J. Itinerant band weak ferromagnetism from the Stoner equations. Eur. J. Phys. 20, 289 (1999).

[b9] Sokolov D. A., Aronson M. C., Gannon W. & Fisk Z. Critical Phenomena and the Quantum Critical Point of Ferromagnetic Zr_1−*x*_Nb_*x*_Zn_2_. Phys. Rev. Lett. 96, 116404 (2006). 16605847 10.1103/PhysRevLett.96.116404

[b10] Zhou J. S. . Critical Behavior of the Ferromagnetic Perovskite BaRuO_3_. Phys. Rev. Lett. 101, 077206 (2008). 18764577 10.1103/PhysRevLett.101.077206

[b11] Wohlfarth E. P. The Theoretical and Experimental Status of the Collective Electron Theory of Ferromagnetism. Rev. Mod. Phys. 25, 211 (1953).

[b12] Moriya T. Theory of itinerant electron magnetism. J. Magn. Magn. Mater. 14, 1 (1979).

[b13] Takahashi Y. On the origin of the Curie-Weiss Law of the Magnetic susceptibility in Itinerant electron Ferromagnetism. J. Phys. Soc. Jpn. 55, 3553 (1986).

[b14] Yu X. Y. . Real-space observation of a two-dimensional skyrmion crystal. Nature (London) 465, 901 (2010). 20559382 10.1038/nature09124

[b15] Rybakov F. N., Borisov A. B., Blugel S. & Kiselev N. S. New Type of Stable Particle-like States in Chiral Magnets. Phys. Rev. Lett. 115, 117201 (2015). 26406851 10.1103/PhysRevLett.115.117201

[b16] Muuhlbauer S. . Skyrmion lattice in a chiral magnet. Science 323, 915 (2009). 19213914 10.1126/science.1166767

[b17] Roβler U. K., Bogdanov A. N. & Pfleiderer C. Spontaneous skyrmion ground states in magnetic metals. Nature (London) 442, 797 (2006). 16915285 10.1038/nature05056

[b18] Caillat T., Fleurial J. P. & Borshchevsky A. Growth and some properties of Cr_11_Ge_19_. J. Alloys Compd. 252, 12–15 (1997).

[b19] Wilson M. N., Butenko A. B., Bogdanov A. N. & Monchesky T. L. Chiral skyrmions in cubic helimagnet films: The role of uniaxial anisotropy. Phys. Rev. B 89, 094411 (2014).

[b20] Voellenkle H., Preisinger A., Nowotny H. & Wittmann A. The crystal structure of Cr_11_Ge_19_, Mo_13_Ge_23_, and V_17_Ge_3_. Z. Kristallog. 124, 9–25 (1967).

[b21] Kolenda M., Stoch J. & Szytula A. Esca and magnetic studies of the Cr-Ge system. J. Magn. Magn. Mater. 20, 99–106 (1980).

[b22] Zagryazhskii V. L., Geld P. V. & Shtolts A. K. Magnetic Susceptibility and Electrical Conductivity of the Highest Chromium Germanide. Sov. Phys. J 11, 23 (1968).

[b23] Pecheur P., Toussaint G., Kenzari H., Malaman B. & Welter R. Ferromagnetism of the chimney-ladder compound Cr_11_Ge_19_. J. Alloys Compd. 262–263, 363–365 (1997).

[b24] Ghimire N. J. . Complex itinerant ferromagnetism in noncentrosymmetric Cr_11_Ge_19_. Phys. Rev. B 85, 224405 (2012).

[b25] Wohlfarth E. P. Thermodynamic aspects of itinerant electron magnetism. Physica B 91, 305–314 (1977).

[b26] Wohlfarth E. P. Very Weak Itinerant Ferromagnets Application to ZrZn_2_. J. Appl. Phys. 39, 1061 (1968).

[b27] Edwards D. M. & Wohlfarth E. P. Magnetic Isotherms in the Band Model of Ferromagnetism. Proc. Roy. Soc. A 303, 127 (1968).

[b28] Arrott A. Criterion for ferromagnetism from observations of magnetic isotherms. Phys. Rev. 108, 1394–1396 (1957).

[b29] Banerjee S. K. On a generalised approach to first and second order magnetic transitions. Phys. Lett. 12, 16–17 (1964).

[b30] Fisher M. E. The theory of equilibrium critical phenomena. Rep. Prog. Phys. 30, 615–730 (1967).

[b31] Stanley H. E. Introduction to Phase Transitions and Critical Phenomena (Oxford University Press, London, 1971).

[b32] Arrott A. & Noakes J. Approximate equation of state for Nickel near its critical temperature. Phys. Rev. Lett. 19, 786 (1967).

[b33] Kaul S. N. Static critical phenomena in ferromagnets with quenched disorder. J. Magn. Magn. Mater. 53, 5–53 (1985).

[b34] Huang K. Statistical Mechanics. 2nd ed. (Wiley, New York, 1987).

[b35] Zhang L. . Critical behavior in the antiperovskite ferromagnet AlCMn_3_. Phys. Rev. B 85, 104419 (2012).

[b36] Zhang L. . Critical properties of the 3D-Heisenberg ferromagnet CdCr_2_Se_4_. Europhys. Lett. 91, 57001 (2010).

[b37] Fan J. Y. . Critical properties of the perovskite manganite La_0.1_Nd_0.6_Sr_0.3_MnO_3_. Phys. Rev. B 81, 144426 (2010).

[b38] Zhang L. . Critical behavior of single crystal CuCr_2_Se_4−*x*_ Br_*x*_ (*x* = 0.25). Appl. Phys. A 113, 201–206 (2013).

[b39] Kouvel J. S. & Fisher M. E. Detailed Magnetic Behavior of Nickel Near its Curie Point. Phys. Rev. 136, A1626 (1964).

[b40] Khan N. . Critical behavior in single-crystalline La_0.67_Sr_0.33_CoO_3_. Phys. Rev. B 82, 064422 (2010).

[b41] Phan M. H. . Tricritical point and critical exponents of La_0.7_Ca_0.3−*x*_Sr_*x*_MnO_3_ (*x* = 0, 0.05, 0.1, 0.2, 0.25) single crystals. J. Alloys Compd. 508, 238–244 (2010).

[b42] Kadanoff L. P. Scaling laws for Ising models near T_*C*_. Physics 2, 263–272 (1966).

[b43] Bhattacharyya A., Jain D., Ganesan V., Giri S. & Majumdar S. Investigation of weak itinerant ferromagnetism and critical behavior of Y_2_Ni_7_. Phys. Rev. B 84, 184414 (2011).

[b44] Perumal A., Srinivas V., Rao V. V. & Dunlap R. A. Quenched disorder and the critical behavior of a partially frustrated system. Phys. Rev. Lett. 91, 137202 (2003). 14525333 10.1103/PhysRevLett.91.137202

[b45] Gerber P. R. Spin-Dimensionality Dependence of Critical Parameters. Z. Physik B 32, 327 333 (1979).

[b46] Fischer S. F., Kaul S. N. & Kronmuller H. Critical magnetic properties of disordered polycrystalline Cr_75_Fe_25_ and Cr_70_Fe_30_ alloys, Phys. Rev. B 65, 064443 (2002).

[b47] Fisher M. E. The renormalization group in the theory of critical behavior, Rev. Mod. Phys. 46, 597–616 (1974).

[b48] Zhang L. . Critical behavior of the single-crystal helimagnet MnSi. Phys. Rev. B 91, 024403 (2015).

[b49] Ghosh K. . Critical phenomena in the double-exchange ferromagnet La_0.7_Sr_0.3_MnO_3_. Phys. Rev. Lett. 81, 4740 (1998).

[b50] Fisher M. E., Ma S. K. & Nickel B. G. Critical exponents for long-range interactions. Phys. Rev. Lett. 29, 917–920 (1972).

[b51] Togawa Y. . Chiral Magnetic Soliton Lattice on a Chiral Helimagnet. Phys. Rev. Lett. 108, 107202 (2012). 22463448 10.1103/PhysRevLett.108.107202

[b52] Koumpouras K., Bergman A., Eriksson O. & Yudin D. A spin dynamics approach to solitonics. Sci. Rep. 6, 25685 (2016). 27156906 10.1038/srep25685 PMC4860584

[b53] Ghimire N. J. . Magnetic phase transition in single crystals of the chiral helimagnet Cr_1/3_NbS_2_. Phys. Rev. B 87, 104403 (2013).

