# Supplementary material for: Anisotropic magnetic coupling with a two-dimensional characteristic in noncentrosymmetric Cr11Ge19
Source: Sci Rep. 2016 Dec 20;6:39338. doi: 10.1038/srep39338 (PMC5171854; doi:10.1038/srep39338)
Supplement: Supplementary Material [file srep39338-s1.pdf]

Supplementary material to " Anisotropic magnetic coupling with a two-dimensional characteristic in noncentrosymmetric $Cr_{11}Ge_{19}$"

Hui Han,[1,2] Lei Zhang,[1,*] Xiangde Zhu,[1] Haifeng Du,[1] Min Ge,[3]

Langsheng Ling,[1] Li Pi,[1,3] Changjin Zhang,[1] and Yuheng Zhang[1,3]

[1]*High Magnetic Field Laboratory, Chinese Academy of Sciences, Hefei 230031,China*

[2]*University of Science and Technology of China, Hefei 230026, China*

[3]*Hefei National Laboratory for Physical Sciences at the Microscale, University of Science and Technology of China, Hefei 230026, China*

Corresponding author. Email: zhanglei@hmfl.ac.cn

I. The characterization of the single crystals

1. The morphology of single crystal $Cr_{11}G_{19}$

The picture of the single crystal of $Cr_{11}Ge_{19}$ has been shown in Fig. S1 (a), which indicates that the size of the single crystals are mm×mm scale. The size of a typical sample in this work is 885×861×680 μm, as depicted in Fig. S1 (b).

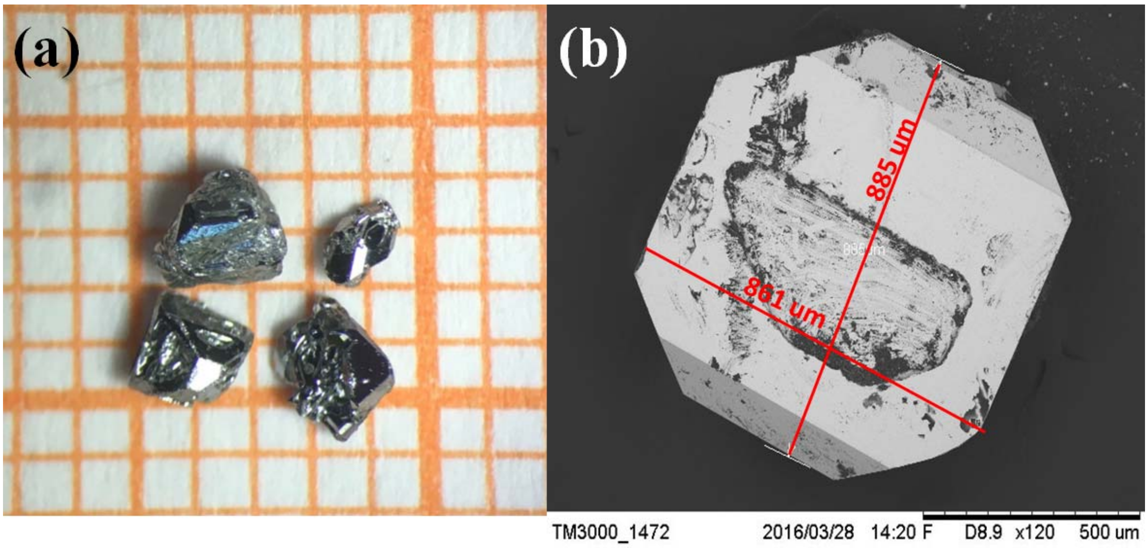

Fig. S1: (a) The photographs of $Cr_{11}Ge_{19}$ single crystals (the scale of the square lattice is 1mm×1mm); (b) the morphology of a typical sample.

2. The EDX spectra at different points on the sample.

We have performed the measurement of EDX spectra at different points on the single crystal sample. The distribution of the elements is shown in Fig S2, which indicates the elements of Cr (red) and Ge (green) are homogeneously distributed in the sample. The proportions of the elements are also shown below in Fig. S3, all of which give that the ratios of Cr : Ge are close to 11 : 19.

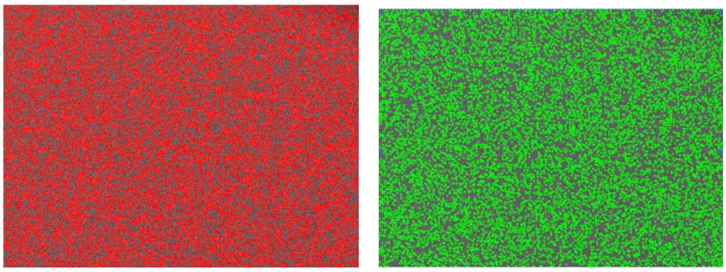

Fig. S2: The distribution of Cr (red) and Ge (red) elements on a scale of 400μm.

项目 1

2016/3/31 20:02:38

谱图处理 :

可能被忽略的峰 : 0.265 keV

处理选项 : 所有经过分析的元素 (已归一化)

重复次数 = 2

标准样品 :

Cr Cr 1-Jun-1999 12:00 AM

Ge Ge 1-Jun-1999 12:00 AM

元素	重量<br>百分比	原子<br>百分比	
Cr K	28.21	35.42	**10.626**
Ge K	71.79	64.58	**19.374**
总量	100.00		

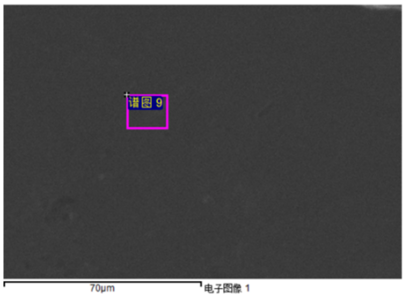

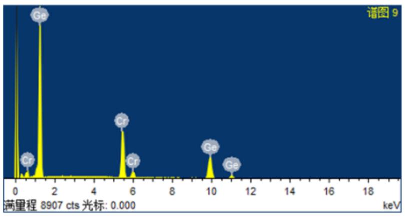

注释:

项目 1

2016/3/31 20:01:00

谱图处理 :

可能被忽略的峰 : 0.265 keV

处理选项 : 所有经过分析的元素 (已归一化)

重复次数 = 2

标准样品 :

Cr Cr 1-Jun-1999 12:00 AM

Ge Ge 1-Jun-1999 12:00 AM

元素	重量<br>百分比	原子<br>百分比	
Cr K	28.66	35.93	**10.779**
Ge K	71.34	64.07	**19.221**
总量	100.00		

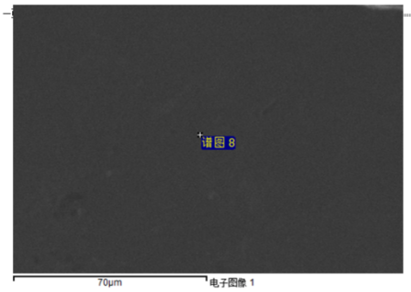

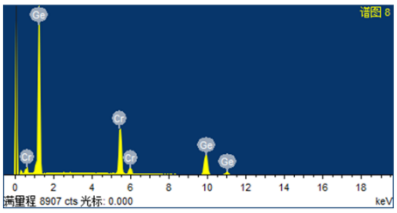

注释:

项目 1

2016/3/31 19:57:42

谱图处理 :

可能被忽略的峰 : 0.266 keV

处理选项 : 所有经过分析的元素 (已归一化)

重复次数 = 2

标准样品 :

Cr Cr 1-Jun-1999 12:00 AM

Ge Ge 1-Jun-1999 12:00 AM

元素	重量百分比	原子百分比	
Cr K	29.56	36.94	**11.082**
Ge K	70.44	63.06	**18.918**
总量	100.00		

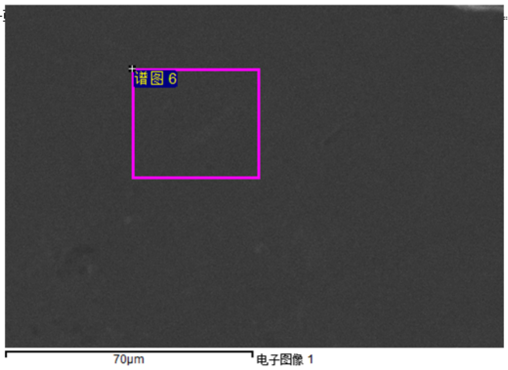

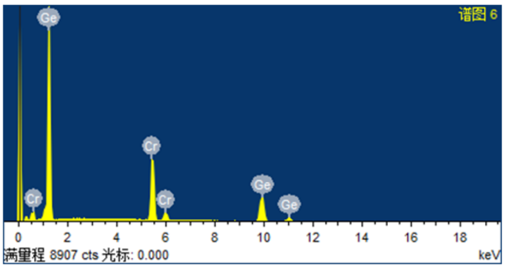

注释：

项目 1

2016/3/31 19:54:55

谱图处理 :

可能被忽略的峰 : 0.265 keV

处理选项 : 所有经过分析的元素 (已归一化)

重复次数 = 2

标准样品 :

Cr Cr 1-Jun-1999 12:00 AM

Ge Ge 1-Jun-1999 12:00 AM

元素	重量百分比	原子百分比	
Cr K	28.48	35.73	**10.719**
Ge K	71.52	64.27	**19.281**
总量	100.00		

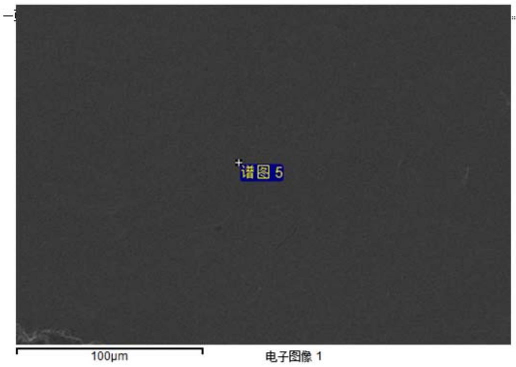

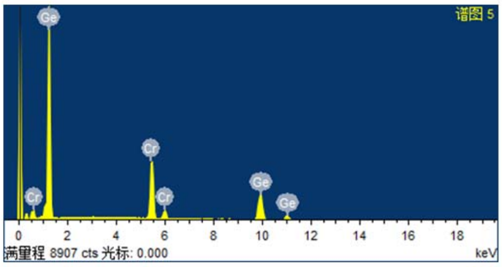

注释：

Fig. S3: The EDX spectra at different points.

II. The Laue photograph

Figure S4. shows the Laue photograph of the bc-plane. All diffraction dots are distributed symmetrically on the two sides of the two main axes. In the reciprocal space, due to the large lattice constant of c, there are much more dots on the c-axis than that on the b-axis on the Laue photograph. Thus, the b- and c-axis can be distinguished. The crystal orientations are marked in the left inset of Fig. 2 (a).

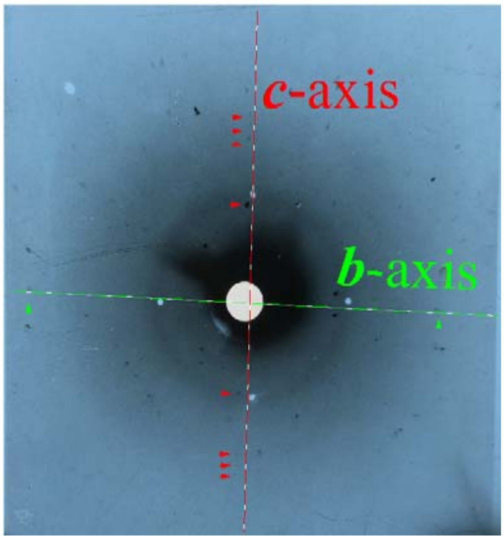

Fig. S4: The Laue photograph of the bc-plane.

III. The angle rotation measurement of magnetization

The angle rotation of the magnetization [M(φ)] was carried out on an angle rotation facility, which is shown in Fig. S5 (a). Figure 5 (b) gives the M(φ) of the bc-plane at 65 K. The b-axis was fixed as the zero point. When measuring the initial isothermal M(H), the applied field H is strictly fixed at the max point on the M(φ) curve, i.e. the c-axis. Then the initial isothermal M(H) measurement was performed. The measurement of the M(H) with H//a, H//b, and H//c was also done similarly.

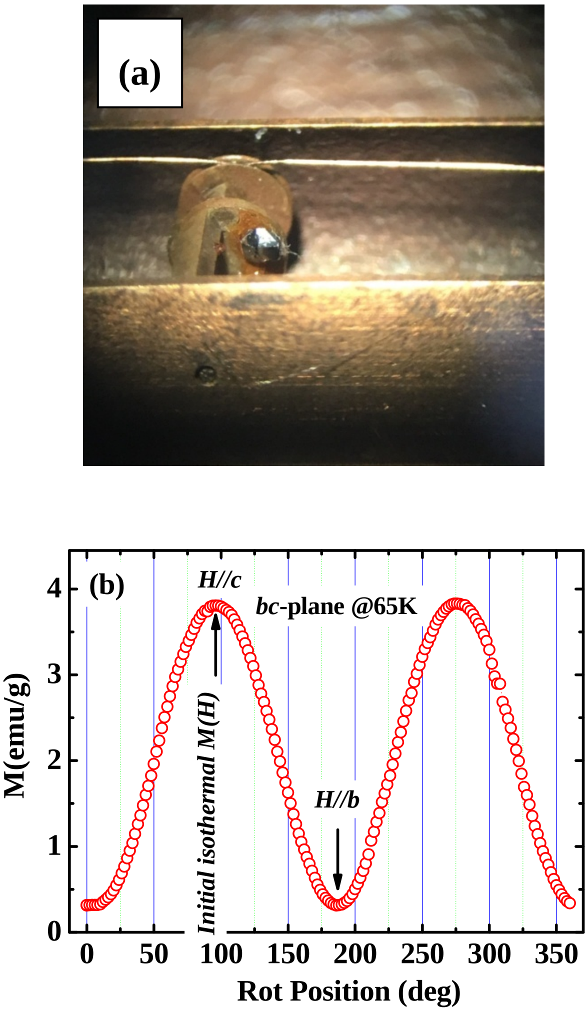

Fig. S5: (a) The sample on the angle rotation facility; (b) the M(φ) of the bc-plane at 65 K. When measuring the isothermal M(H), the applied field is strictly fixed at the max point on the M(φ) curve.
